# Supplementary material for: Analysis of Neisseria gonorrhoeae and Mycoplasma genitalium from nucleic acid amplification test specimens, Nunavut region of Inuit Nunangat, Canada, 2020–2023
Source: Microbiol Spectr. 2025 Aug 13;13(10):e01553-25. doi: 10.1128/spectrum.01553-25 (PMC12502515; doi:10.1128/spectrum.01553-25)
Supplement: Supplemental tables and figure — Tables S1 and S2, and Fig. S1. [file spectrum.01553-25-s0001.docx]

| **Year** | **Sex** | **Region** | | | **Total by sex** | **Total** |
| --- | --- | --- | --- | --- | --- | --- |
|  |  | **Kitikmeot** | **Kivalliq** | **Qikiqtaaluk** |  |  |
| **2020** | Male | 11 | 71 | 221 | 303 | 725 |
|  | Female | 19 | 94 | 309 | 422 |  |
|  | Not Given | 0 | 0 | 0 | 0 |  |
| **2021** | Male | 0 | 24 | 97 | 121 | 355 |
|  | Female | 0 | 65 | 158 | 223 |  |
|  | Not Given | 7 | 2 | 2 | 11 |  |
| **2022** | Male | 0 | 51 | 105 | 156 | 523 |
|  | Female | 0 | 93 | 187 | 280 |  |
|  | Not Given | 80 | 4 | 3 | 87 |  |
| **2023** | Male | 0 | 116 | 99 | 215 | 618 |
|  | Female | 0 | 155 | 149 | 304 |  |
|  | Not Given | 99 | 0 | 0 | 99 |  |
| **Total** | | **216** | **675** | **1330** | **2221** | |

**Table S1.** Sex and geographic distribution of *Neisseria gonorrhoeae*-positive NAAT specimens sent to NML from Nunavut, Canada, 2020-2023

**Figure S1.** *Neisseria gonorrhoeae* Multi-Antigen Sequence Type distribution of the 41 most common sequence types by region in Nunavut, Canada, 2020-2023

**Table S2.** Predicted *Neisseria gonorrhoeae* antimicrobial resistance rates for NAATs by region in Nunavut, Canada, 2020-2023

| **Resistance Category** | **MICs (mg/L)** | **Kitikmeot** | **Kivalliq** | **Qikiqtaaluk** | **Overall** |
| --- | --- | --- | --- | --- | --- |
| Intermediate to decreased susceptibilty to cephalosporins^a^ | ≥0.032 - 0.125 (Ceftriaxone) | 49.07% | 27.85% | 10.68% | 19.63% |
|  | ≥0.032 - 0.25 (Cefixime) |  |  |  |  |
| Ciprofloxacin resistant^b^ | ≥1 | 58.80% | 55.26% | 37.59% | 45.02% |
| Azithromycin resistant^c^ | ≥1 | 3.24% | 8.30% | 11.35% | 9.64% |
| **Total** | | **216** | **675** | **1330** | **2221** |
| Specimens were predicted to have this resistant category if:   1. At least three SNPs of *ponA* L421P, *mtrR* delA, *porB* G120/A121 or *penA* A501/N513Y/G543S were detected 2. Any SNP of *gyrA* S91 or *parC* D86/S87/S88 was detected 3. Any SNP of the *mtrR* promoter or 23S rRNA A2059G/C2611T was detected | | | | | |
